# Agreement and reproducibility of intraoral scan- versus CBCT-based three-dimensional implant position assessment in the posterior mandible — a paired in vitro method-comparison study

**DOI:** 10.1186/s40729-026-00725-y

**Published:** 2026-10-23

**Authors:** Georg Hoene, Shabnam Shahpasand, Felix Karl Waffenschmidt, Frederik Holdorf, Robert Stünkel, Alexander-Nicolai Zeller, Philipp Kauffmann

**Affiliations:** 1 Clinic for Oral and Maxillofacial Surgery, University Medical Center Goettingen, Robert-Koch-Strasse 40, D-37075 Goettingen, Germany; 2 Department of Phoniatrics and Pediatric Audiology, University Hospital Muenster, Muenster, Germany; 3 Dental Practice, Hildesheim, Germany; 4 Dental Practice, Aachen, Germany

**Keywords:** Cone-beam computed tomography, Intraoral scanner, Bland-Altman analysis, Implant position accuracy, Digital workflow, In vitro study

## Abstract

**Background:**

Three-dimensional postoperative implant assessment is increasingly performed using intraoral scan (IOS) superimposition as a radiation-free alternative to cone-beam computed tomography (CBCT). However, it remains unclear to what extent IOS- and CBCT-based measurements agree and how reproducibility changes across posterior mandibular configurations.

**Methods:**

In this paired in vitro method-comparison study, based on the experimental platform of a previous study, 60 implants placed at Fédération Dentaire Internationale (FDI) positions 35, 36, 45, 46 and 47 in bounded edentulous spaces (BES) and distal extension situations (DE) underwent three IOS-based and three CBCT-based postoperative assessments relative to the virtual plan using the Treatment Evaluation Tool of the planning software. Angular, mesiodistal, buccolingual, apico-coronal, and three-dimensional (3D) deviations at implant base and apex were recorded, resulting in 180 paired measurements. Agreement was assessed by Bland-Altman analysis, and within-method reproducibility by the within-implant standard deviation (Sw). IOS variability was additionally separated into within-scan and between-scan components. Non-parametric analyses were used for comparisons by indication and FDI position.

**Results:**

Mean absolute IOS–CBCT differences were 0.73° ± 0.55° for angular deviation, 0.22 ± 0.20 mm for the 3D base offset and 0.26 ± 0.22 mm for the 3D apical offset. Bland-Altman bias (CBCT − IOS) was small (− 0.35°; −0.05 mm; −0.07 mm) with 95% limits of agreement of [− 2.01°; +1.31°], [− 0.62; +0.53] mm and [− 0.73; +0.58] mm. Within-method reproducibility was superior for IOS in 8 of 9 parameters (e.g., Sw 0.20° vs. 0.55° for the angle), and IOS variability was dominated by scan-to-scan rather than within-scan re-evaluation. Discrepancies were larger in DE than in BES sites in 7 of 9 parameters (all *p* < 0.001 for the principal apical and buccolingual components) and were larger at position 47 than at position 35 (3D base *p* = 0.004; 3D apex *p* = 0.011).

**Conclusions:**

In this in vitro setting, IOS- and CBCT-based implant assessment in the posterior mandible showed good agreement with small overall differences, and IOS demonstrated lower within-method variability than CBCT. IOS-based superimposition may therefore serve as a radiation-free complement to CBCT for the postoperative evaluation of single implants in bounded posterior sites. Agreement decreased in distal extension situations and at more distal positions; in these configurations, and whenever clinical decisions depend on sub-millimeter or sub-degree precision, CBCT remains the reference technique. Confirmation in vivo is required before routine clinical use.

## Background

Dental implant rehabilitation has evolved into a digitally driven, multistep workflow in which virtual planning, computer-assisted insertion and postoperative verification are increasingly interlinked [1, 2]. While considerable attention has been devoted to the transfer of the virtual plan into the patient — through static surgical guides or dynamic navigation systems — the final step of the workflow, the verification of the achieved three-dimensional implant position, has received comparatively less methodological scrutiny [3, 4]. Knowledge of the true postoperative position is nonetheless clinically relevant: it informs prosthetic planning, allows the surgeon to recognize deviations that may compromise biomechanics or adjacent anatomical structures, and provides the evidentiary basis on which guided and navigated protocols are compared [2, 5].

The current clinical reference method for postoperative position assessment is cone-beam computed tomography (CBCT) [6]. CBCT provides volumetric data that can be registered to the original treatment plan and is widely used in studies of static and dynamic implant placement [7, 8]. However, CBCT acquisition exposes the patient to ionizing radiation, is subject to metallic artifacts in the presence of implants and restorations, and entails additional cost and infrastructure [6, 9]. In line with the ALARA (as low as reasonably achievable) principle, repeated CBCT acquisition purely for verification purposes is increasingly difficult to justify whenever a comparable diagnostic yield can be achieved by a radiation-free modality [6, 10].

Intraoral optical scanning (IOS) has emerged as a candidate for this role. Combined with implant-specific scan bodies, an IOS can capture the three-dimensional position and orientation of an inserted implant, and the resulting surface mesh can be aligned to the virtual plan in dedicated planning software [11–13]. Several in vitro and clinical studies have compared IOS-based implant position assessment with CBCT-based assessment and have reported deviations in the sub-millimeter range for the implant base and apex, and angular deviations below 1° to 2° under standardized conditions [8, 12–14]. In most of these designs, the preoperative CBCT-based virtual plan serves as the common reference: the deviations of the postoperative CBCT- and IOS-based reconstructions from this plan are determined separately and then compared, frequently with the CBCT-derived deviation regarded as the reference value.

Regarding CBCT as the reference is, however, problematic. In a clinical or experimental scenario in which an implant has already been inserted into bone or into a standardized model, the true three-dimensional position of the implant is not directly accessible. Both CBCT- and IOS-based evaluations rely on segmentation, surface or volumetric matching and operator-dependent landmark identification, each of which contributes its own measurement uncertainty [9, 11, 15]. Without an independent gold standard, neither modality can be designated as the more accurate one. What can be quantified is (i) the agreement between the two methods, i.e., the magnitude and systematic component of their differences, and (ii) the within-method reproducibility of each modality under repeated acquisition and evaluation [16, 17]. These two properties — agreement and reproducibility — determine whether a radiation-free modality such as IOS can complement or, in selected situations, replace CBCT in clinical verification, irrespective of which modality is closer to the true implant position.

The present in vitro study addresses this methodological gap. It builds on a previously published experimental platform, in which 60 dental implants were inserted into 12 standardized partially edentulous mandibular models using either a static surgical guide or a dynamic navigation system, and in which insertion accuracy relative to the virtual plan was reported using postoperative CBCT [18]. The same physical models, virtual plans and insertions are reused here. In contrast to the original study, the postoperative implant position is evaluated by both CBCT and IOS, and the two evaluations are compared without the assumption that either method represents the truth.

The objectives of this study are therefore:

1. To quantify the agreement between IOS-based and CBCT-based postoperative implant position assessment at the implant base, the implant apex and in angular terms, using Bland-Altman analysis (mean difference and limits of agreement).
2. To quantify the within-method reproducibility of each modality under repeated evaluation (CBCT) and repeated scanning and evaluation (IOS) by the same examiner.
3. To explore whether agreement and reproducibility are influenced by Fédération Dentaire Internationale (FDI) tooth position (35, 36, 45, 46, 47) and by the clinical situation — bounded edentulous space (BES) versus distal extension (DE).

No claim is made regarding the absolute accuracy of either modality with respect to the true implant position.

## Materials & methods

### Study design

This in vitro method-comparison study compared two postoperative methods for three-dimensional verification of dental implant position: cone-beam computed tomography (CBCT) and digital surface acquisition with an intraoral scanner (IOS). Because no independent ground truth for the true implant position is available in the present setup, neither modality is treated as a reference standard; the analysis instead quantifies the agreement between the two methods and the internal reproducibility of each.

The experimental platform — mandibular models, virtual planning, implant insertions, surgical guides, and dynamic navigation — was generated within a previous study by our group that compared the intraoperative accuracy of static and dynamic guidance [18]. The present work uses the same physical models and implants to address a different research question: whether postoperative implant position derived from an intraoral surface scan can serve as a radiation-free complement to CBCT-based evaluation. Insertion accuracy with respect to the virtual plan has been reported previously [18] and is not re-analyzed here; all IOS-based measurements, the IOS-versus-CBCT comparisons, the repeated-evaluation reproducibility analyses, and the Bland-Altman analyses are original to the present study.

A total of 60 implants in 12 partially edentulous mandibular models were evaluated. The sample size was not derived from a formal a priori power analysis; a sample of 60 implants distributed over 12 models is in line with comparable in vitro investigations of implant position accuracy [8, 14, 18].

### Specimens and implants

Twelve identical partially edentulous mandibular training models (Mandibula teilbezahnt, Art. No. 1009; GOS, Northeim, Germany), manufactured from a proprietary polymer blend simulating type D1 bone, were used. Each model was dentate at the mandibular left second molar (37), from the mandibular left first premolar through the mandibular right first premolar (34–44), and contained two standardized edentulous areas: a single-span bounded edentulous space (BES) in the third quadrant (sites 35 and 36) and a distal extension (DE) situation in the fourth quadrant (sites 45, 46 and 47).

**Fig. 1:**
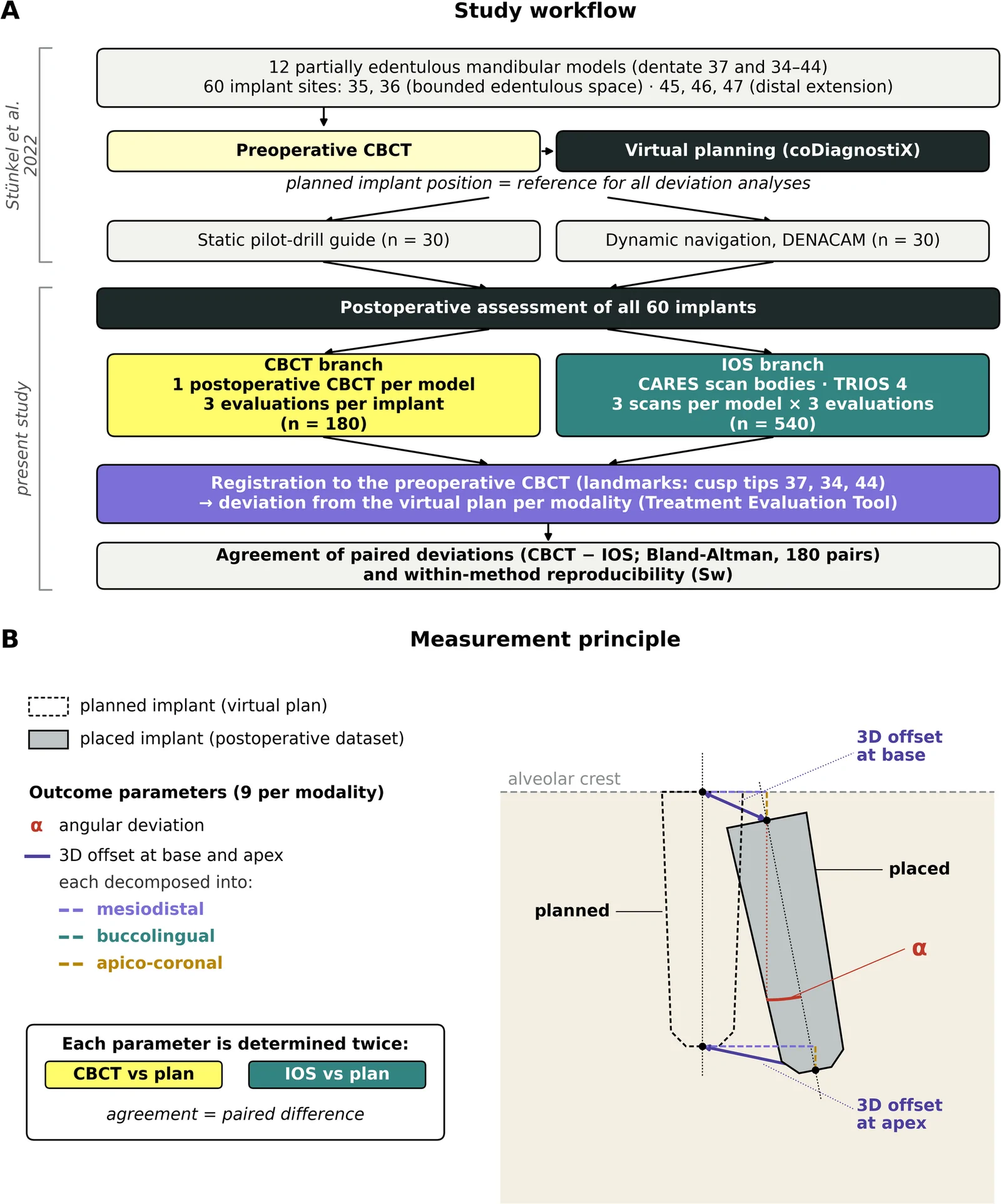
Workflow and measurement principle. (**A**) Study workflow. Models, preoperative CBCT, virtual planning and implant insertion were generated within a previous study [18]; all postoperative assessments and analyses are original to the present work. (**B**) Schematic definition (not to scale) of the deviation parameters between the planned and the placed implant — angular deviation, three-dimensional offset and its mesiodistal, buccolingual and apico-coronal components at the implant base and apex — adapted from prior work [21]. All parameters were determined separately in the CBCT- and IOS-based reconstructions relative to the same virtual plan; the buccolingual component is perpendicular to the image plane and therefore not depicted. CBCT, cone-beam computed tomography; IOS, intraoral scan; Sw, within-implant standard deviation; 3D, three-dimensional

Sixty bone-level implants (Straumann Bone Level Implant, Ø 4.1 mm RC, length 10 mm; Institut Straumann AG, Basel, Switzerland) were placed at FDI positions 35, 36, 45, 46 and 47 — five implants per model. The overall workflow and the geometric basis for the deviation parameters are summarized in Fig. 1.

### Imaging and virtual planning

Preoperative and postoperative CBCT scans were acquired for every model with a single device (PaX-Zenith 3D; Vatech, Hwaseong, South Korea) using identical exposure parameters: tube voltage 120 kV, tube current 6 mA, exposure time 24 s, dose-area product 18.37 dGy·cm², a field of view of 160 × 160 mm and a voxel size of 0.3 mm. Models were oriented parallel to the occlusal plane and centered at 15 cm above the receptor.

For dynamic navigation, an intraoral fiducial marker (DENAMARK; mininavident AG, Liestal, Switzerland) was secured to the anterior teeth of each model with thermoplastic resin via a reusable tray (DENATRAY 01 M1600). Preoperative Digital Imaging and Communications in Medicine (DICOM) datasets were imported into the planning software (coDiagnostiX, version 10.5; Dental Wings, Chemnitz, Germany). The mandibular canal was annotated, and each implant was virtually positioned individually with respect to angulation and the mesiodistal, buccolingual and apico-coronal axes, ensuring adequate inter-implant and bone-margin distances. Final positions were verified in sagittal, axial and coronal multiplanar reconstructions and on the three-dimensional reconstruction.

For half of the implants, a tooth-supported surgical guide was generated. A stone cast of each model was digitized by the collaborating dental laboratory using a desktop laboratory scanner (3Shape A/S, Copenhagen, Denmark), and the resulting surface dataset was registered to the corresponding preoperative CBCT dataset in coDiagnostiX by selecting three congruent anatomical landmarks; the registration was verified visually in the multiplanar and three-dimensional views before guide export. Guides were three-dimensionally printed in a biocompatible photopolymer (MED610) on a Connex Objet500 printer (Stratasys, Rheinmünster, Germany). Universal sleeves (steco-system-technik, Hamburg, Germany) with an internal diameter 0.15 mm larger than the 2.2-mm pilot drill and a vertical extension of 6 mm were inserted into the guide; inspection windows were incorporated to verify passive seating on the residual dentition.

### Implant insertion

All implant placements were performed on dental simulation units (DSEclinical 5197 KaVo Combination Unit; KaVo Dental, Biberach, Germany) equipped with a patient-simulator dummy and a facial mask restricting access to the oral cavity, thereby reproducing clinically realistic ergonomic constraints. The manufacturer’s drilling protocol (Straumann) was followed throughout: cortical perforation with a round bur, sequential pilot drilling at 800 rpm with the 2.2-mm pilot drill 1 and the 2.8-mm pilot drill 2 to a depth of 10 mm, followed by the 3.5-mm spiral drill PRO. Fine preparation comprised the BL profile drill and the BL tap (Straumann). After visual inspection of the osteotomy, implants were inserted at 15 rpm with the Loxim transfer device, and final seating was confirmed with a torque ratchet according to the manufacturer’s specifications.

Two operators performed the implant placements. Operator 1 placed all 30 implants in models 1–6, and operator 2 placed all 30 implants in models 7–12. Insertion modality was assigned per model in a non-randomized, balanced fashion: models 1–3 and 7–9 (*n* = 30 implants) were treated with the static three-dimensionally printed surgical guide, and models 4–6 and 10–12 (*n* = 30 implants) with dynamic optical navigation. Both operators thus performed both insertion modalities in equal proportion.

#### Static guided surgery

Thirty implants were inserted with the three-dimensionally printed tooth-supported guide. The guide was seated on the model and its passive fit verified through the inspection windows. The 2.2-mm pilot osteotomy was performed through the guide sleeve. Subsequent osteotomies and implant insertion followed the Straumann drilling protocol described above without further guidance, on the assumption — supported by previous data — that pilot-drill guidance is sufficient to constrain the trajectory of the subsequent freehand steps [18].

#### Dynamic navigation

Thirty implants were inserted under real-time optical navigation (DENACAM^®^ system; mininavident^®^ AG, Liestal, Switzerland). The system comprises a computing unit (DENACOMP), a touch-screen display (DENATOUCH), the intraoral marker (DENAMARK) mounted on a tray (DENATRAY), a calibration block (DENAREG), a contra-angle adapter (DENADAPT), and a stereo camera (DENACAM). Positional accuracy is established through three sequential optical registrations: marker-to-camera, camera-to-drill, and marker-to-CBCT dataset. The cameras were factory-calibrated at operating temperature using a defined three-dimensional point pattern; the proprietary line pattern of the intraoral marker provides intraoperative reference. Each drill was registered in the corresponding slot of DENAREG before use, allowing real-time overlay of drill-tip position on the preoperative CBCT throughout osteotomy preparation and implant insertion.

### Postoperative evaluation

For every model, two types of postoperative datasets were acquired: one postoperative CBCT (identical exposure parameters to the preoperative scan) and three intraoral surface scans. Both datasets were compared with the original virtual plan using the Treatment Evaluation Tool of coDiagnostiX (version 10.5). The geometric definition of the resulting deviation parameters — angular deviation, three-dimensional offsets at implant base and apex, and the buccolingual, mesiodistal and apico-coronal components — is illustrated in Fig. 1B.

For each model, both evaluations were performed by the same operator who had placed the implants — that is, operator 1 evaluated models 1–6 and operator 2 evaluated models 7–12. Both examiners were blinded to the insertion modality (static guide vs. dynamic navigation) at the time of evaluation. All evaluations were carried out within a single session per model, without a formal washout period between repeated measurements.

#### CBCT-based evaluation

Each postoperative CBCT was registered to the corresponding preoperative dataset by manual selection of three predefined congruent anatomical landmarks — the cusp tips of the mandibular left second molar (37) and of the mandibular left and right first premolars (34 and 44) — which were identical for all models and for both DICOM–DICOM and DICOM–STL (standard tessellation language) registration. Every registration was inspected in the axial, sagittal and coronal reconstructions and in the three-dimensional view; registrations with visible misalignment were repeated before the deviation analysis. The postoperative implant axis was then aligned on the multiplanar reconstruction. The Treatment Evaluation Tool computed the angular deviation (degrees) and the linear deviations (millimeters) between planned and actual implant position at the implant base and apex, decomposed into three-dimensional offset and into the buccolingual, mesiodistal and apico-coronal components. To assess intra-examiner reproducibility, each postoperative CBCT (one per model) was evaluated three times by the same examiner.

#### IOS-based evaluation

For optical evaluation, RC Mono Scan Bodies (CARES; Ø 4.1 mm, height 10 mm, polyetheretherketone/titanium–aluminum–niobium alloy; Straumann) were hand-tightened onto each implant to a maximum of 10 Ncm and remained in place throughout all three repeated scans of a given model. Surface scans were acquired with an intraoral scanner (TRIOS 4, system S3P-2, software version 22.1; 3Shape A/S, Copenhagen, Denmark) on the same simulation units used for implantation, with the facial mask in place to replicate clinical access conditions. Prior to each session, the scanner was color-calibrated according to the manufacturer’s instructions. Scanning followed the manufacturer-recommended sequence: occlusal, lingual, then buccal. Areas of insufficient capture were re-scanned and merged automatically by the software.

Each model was scanned three times, and each of the three resulting STL datasets was subsequently evaluated three times by the same examiner, yielding 108 model-level IOS evaluation runs (12 models × 3 scans × 3 evaluations) and a corresponding 540 implant-level IOS measurements across the 60 implants (3 scans × 3 evaluations per implant). For the agreement analyses, the first evaluation of each scan was paired with the correspondingly numbered CBCT evaluation (scan k with CBCT evaluation k), providing 180 paired IOS–CBCT assessments (60 implants × 3); all IOS evaluations were used for the reproducibility analyses. The STL datasets were registered to the preoperative DICOM volume using the same three landmarks and the same visual verification as for the DICOM–DICOM registration. Scan bodies were detected semi-automatically by the Treatment Evaluation Tool and assigned to their respective implant sites. The same set of angular and linear deviation metrics described for the CBCT-based evaluation was computed.

### Statistical analysis

All statistical analyses were performed in R using RStudio (version 2023.06.0 + 421; Posit, Boston, MA, USA). The structured dataset comprised, for every implant, the angular deviation, the three-dimensional offset at base and apex, and the buccolingual, mesiodistal and apico-coronal components at base and apex, each expressed as the absolute difference between IOS- and CBCT-derived measurements; for Bland-Altman analysis, signed differences were calculated as CBCT minus IOS. Within-method reproducibility was expressed as the within-implant standard deviation (Sw), calculated as the pooled within-implant standard deviation across all repeated assessments of the same implant by the same modality. For IOS, total Sw was additionally decomposed into a within-scan component (Sw_within-scan; three evaluations of the same scan) and a between-scan component (Sw_between-scan; three independent scans).

Distribution was assessed with the Shapiro-Wilk test and homogeneity of variance with Levene’s test. Normally distributed variables were compared with Welch’s t test (two groups) or one-way analysis of variance (more than two groups); non-normally distributed variables with the Mann-Whitney U test or the Kruskal-Wallis test. Descriptive statistics are reported as mean ± standard deviation. The level of statistical significance was set at *p* < 0.05.

Three levels of comparison were performed: (i) pairwise comparisons of individual FDI implant sites (e.g., 35 vs. 45, 36 vs. 46, 35 vs. 47); (ii) regional comparisons of the bounded edentulous space (35/36) versus the distal extension (45/46/47); and (iii) within-method reproducibility analyses, comparing the nine IOS evaluation runs per model (three scans × three evaluations) and the three repeated CBCT evaluations per model. Bland-Altman analysis was used to quantify systematic bias and 95% limits of agreement between IOS and CBCT for all nine parameters.

## Results

### Study sample and analytical design

A total of 180 implant site assessments were available for paired analysis, derived from 60 implants placed in the posterior mandible at FDI positions 35, 36, 45, 46 and 47, in bounded edentulous spaces (BES) and distal extension situations (DE). Each implant was assessed in three intraoral scans (IOS) and in three repeated evaluations of the postoperative cone-beam computed tomography (CBCT) using the Treatment Evaluation Tool of coDiagnostiX, yielding paired IOS–CBCT readings for nine geometric parameters per implant: angular deviation, three translational components (mesiodistal, buccolingual, apico-coronal) at the implant base and at the apex, as well as three-dimensional (3D) offsets at the base and at the apex. The overall measurement workflow and the underlying geometric principle are summarized in Fig. 1.

### Overall agreement between IOS- and CBCT-based assessment

**Fig. 2:**
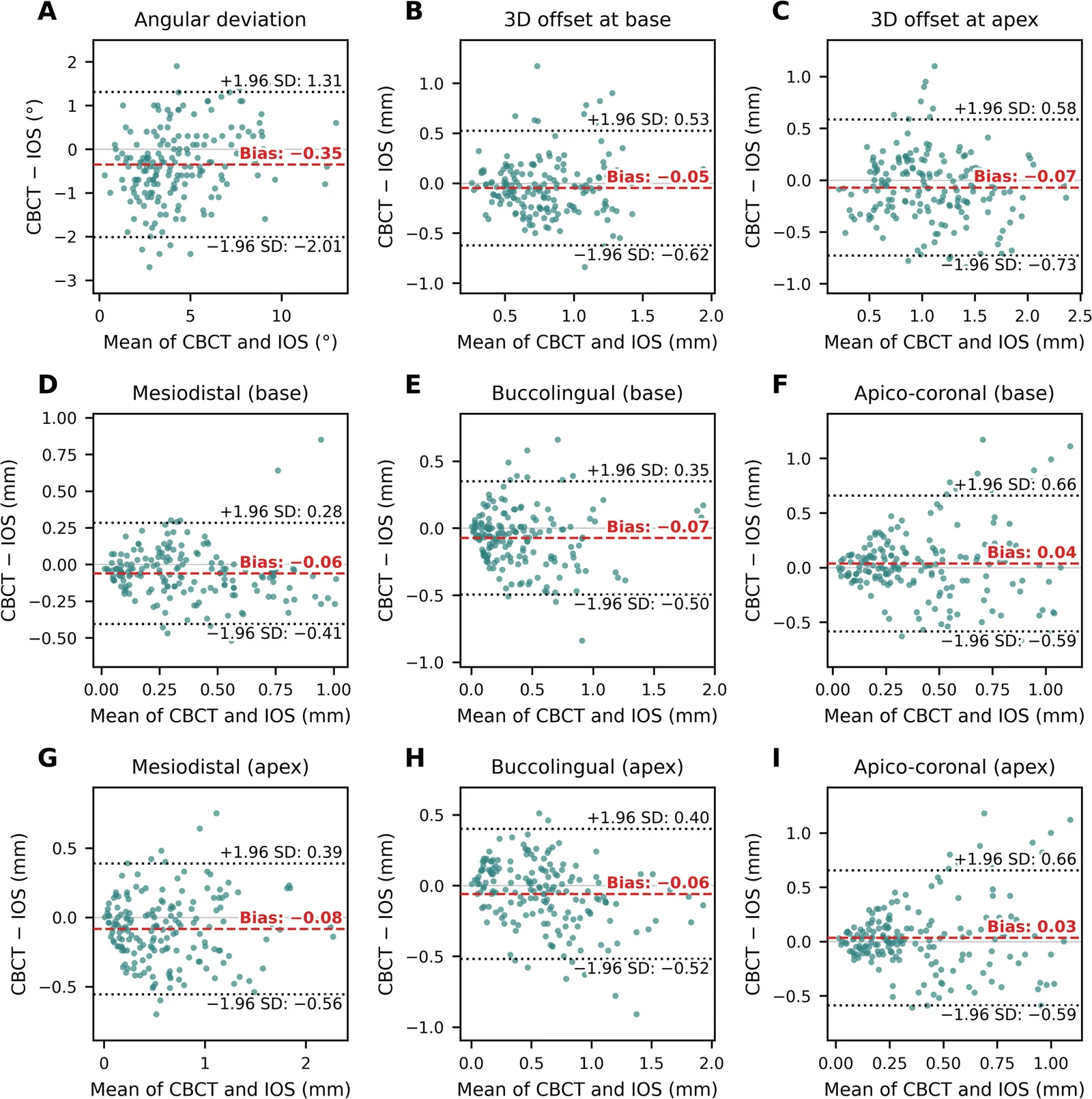
Bland-Altman plots of the paired CBCT- and IOS-based measurements (*n* = 180 pairs) for (**A**) angular deviation, (**B**, **C**) the 3D offset at the implant base and apex and (**D**–**I**) the mesiodistal, buccolingual and apico-coronal components at the base and apex. Differences are plotted as CBCT minus IOS against the mean of both methods; negative values indicate larger deviations from the virtual plan in the IOS-based assessment. Red dashed line, mean difference (bias); dotted lines, 95% limits of agreement (bias ± 1.96 × SD of the differences). CBCT, cone-beam computed tomography; IOS, intraoral scan; 3D, three-dimensional; SD, standard deviation

Bland-Altman analysis (Fig. 2) showed that the systematic bias between modalities (CBCT − IOS) was small: −0.35° for angular deviation, − 0.05 mm for the 3D base offset and − 0.07 mm for the 3D apical offset; negative values indicate slightly larger deviations from the virtual plan in the IOS-based assessment. The corresponding 95% limits of agreement spanned [− 2.01°; +1.31°] for the angle, [− 0.62; +0.53] mm at the base and [− 0.73; +0.58] mm at the apex. Overall, 75.0% of the absolute angular differences were ≤ 1°, and 91.1% and 85.0% of the absolute 3D offset differences at the base and apex, respectively, were ≤ 0.5 mm.

**Fig. 3:**
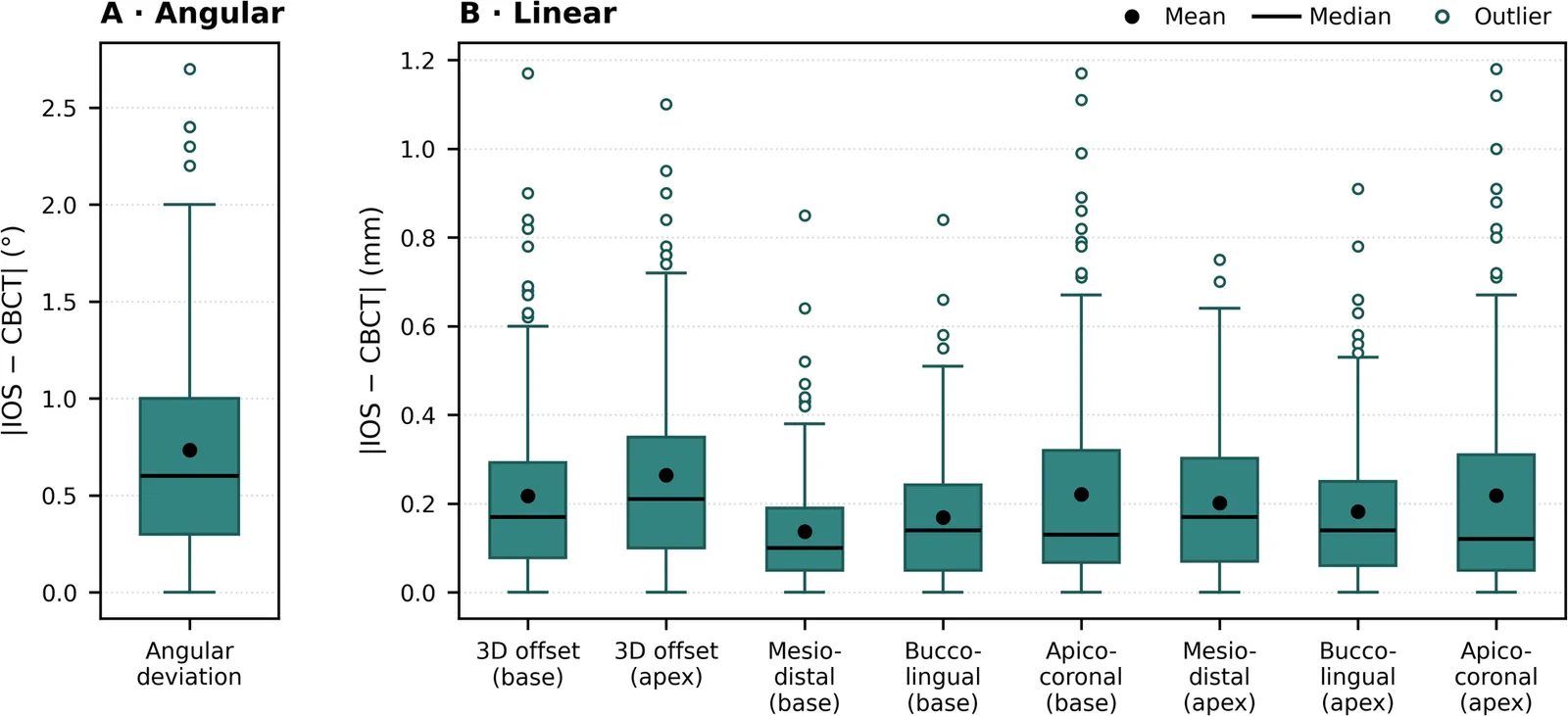
Absolute differences between IOS- and CBCT-based measurements for (**A**) angular deviation and (**B**) the eight linear parameters across all 180 paired assessments (60 implants × 3). Boxes, interquartile range; horizontal line, median; filled circle, mean; whiskers, 1.5 × interquartile range; open circles, outliers. CBCT, cone-beam computed tomography; IOS, intraoral scan; 3D, three-dimensional

Across all 180 paired assessments, the absolute differences between the two modalities were small (Fig. 3). The mean angular discrepancy was 0.73° ± 0.55° (range 0–2.7°), and three-dimensional positional differences averaged 0.22 ± 0.20 mm at the implant base and 0.26 ± 0.22 mm at the apex. Translational components were of comparable magnitude in all three axes, with mean values between 0.14 and 0.22 mm.

### Within-method reproducibility

**Fig. 4:**
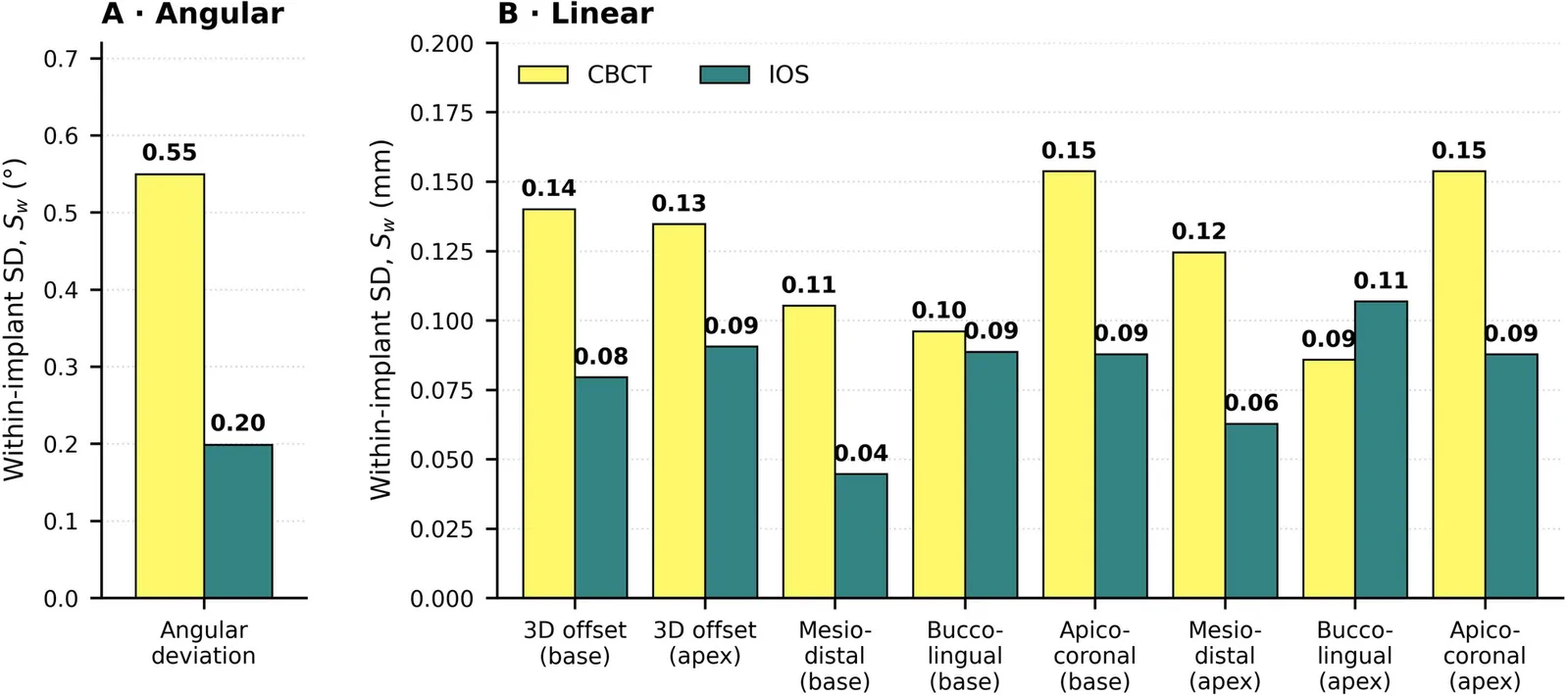
Within-method reproducibility, expressed as the pooled within-implant standard deviation (Sw) across replicates, for (**A**) angular deviation and (**B**) the eight linear parameters. CBCT: three repeated evaluations of one postoperative CBCT per model; IOS: three scans per model with three evaluations per scan. Lower values indicate higher reproducibility. CBCT, cone-beam computed tomography; IOS, intraoral scan; 3D, three-dimensional

Within-method reproducibility, expressed as the pooled within-implant standard deviation (Sw) across repeated assessments, was consistently lower for IOS than for CBCT in eight of nine parameters (Fig. 4). For angular deviation, Sw was 0.55° for CBCT and 0.20° for IOS, corresponding to an approximately 2.8-fold improvement. Similar patterns were observed for the remaining translational and 3D parameters at the implant base and apex. The single exception was the buccolingual component at the apex, where Sw was 0.09 mm for CBCT and 0.11 mm for IOS. Non-parametric comparison across the three repeated CBCT and IOS evaluations confirmed that intra-method dispersion did not differ systematically between repetitions for either modality (data not shown). Decomposition of the IOS reproducibility showed that the within-scan component (Sw_within-scan; three evaluations of the same scan) was minimal — for example, ≤ 0.02° for the angle — whereas the between-scan component (Sw_between-scan; three independently acquired scans) accounted for the majority of total IOS dispersion. The dominant source of IOS variability was thus scan acquisition rather than digital re-evaluation.

### Effect of edentulous gap type

**Fig. 5:**
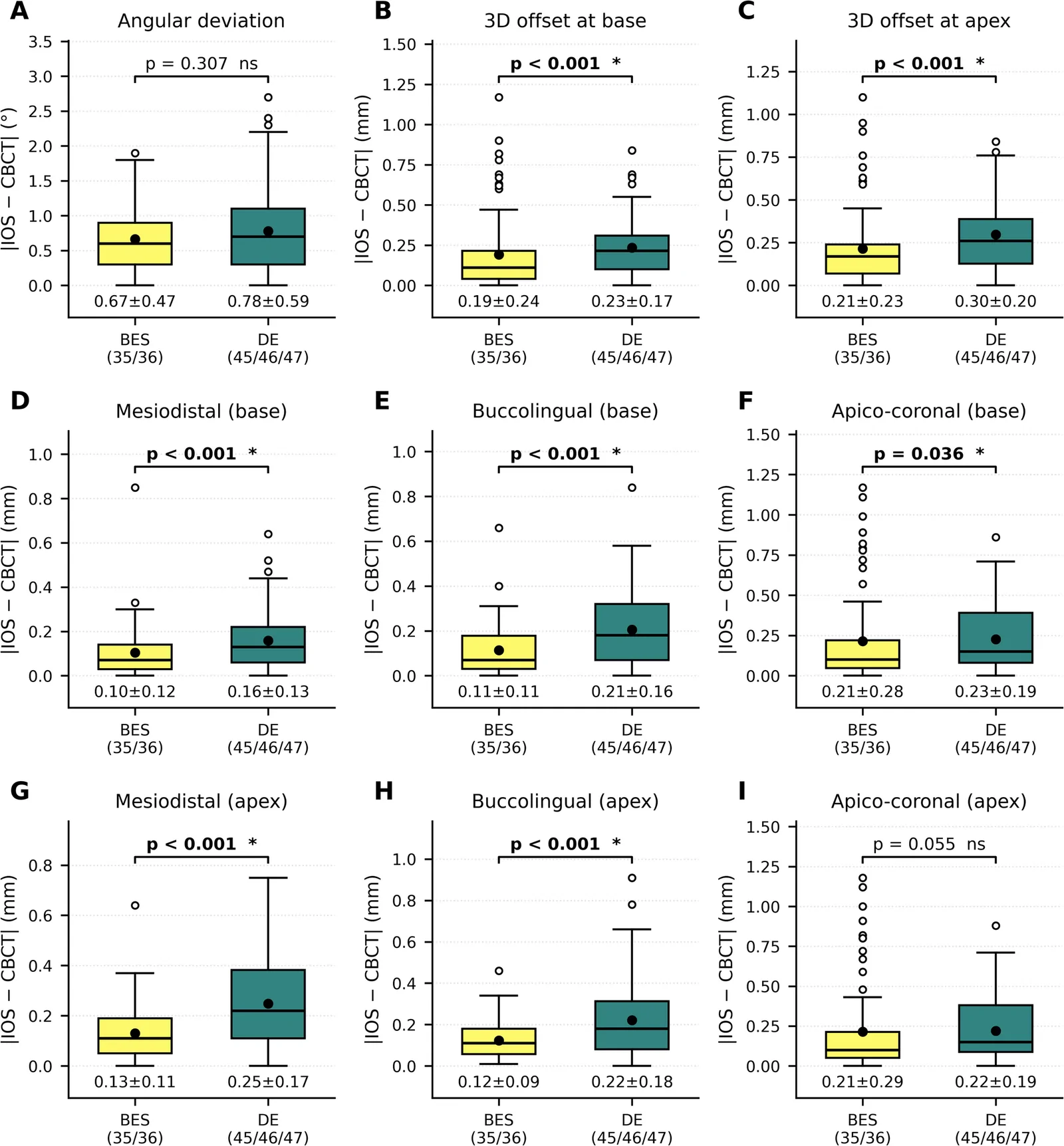
Absolute IOS–CBCT differences in bounded edentulous spaces (BES; positions 35 and 36; *n* = 72) and distal extension situations (DE; positions 45–47; *n* = 108) for all nine parameters (**A**–**I**). Values below the boxes are mean ± SD. P values from the Mann-Whitney U test; *, *p* < 0.05; ns, not significant. Box plot elements as in Fig. 3. CBCT, cone-beam computed tomography; IOS, intraoral scan; SD, standard deviation; 3D, three-dimensional

Stratification by indication (Fig. 5) revealed larger IOS–CBCT discrepancies in distal extension sites compared with bounded edentulous spaces in seven of nine parameters. Differences were most pronounced at the implant apex: the mesiodistal apex difference was 0.13 ± 0.11 mm in BES versus 0.25 ± 0.17 mm in DE (*p* < 0.001), the buccolingual apex difference was 0.12 ± 0.09 mm versus 0.22 ± 0.18 mm (*p* < 0.001), and the 3D apical offset was 0.21 ± 0.23 mm versus 0.30 ± 0.20 mm (*p* < 0.001). Comparable differences emerged at the implant base, for example in the buccolingual component (0.11 ± 0.11 mm vs. 0.21 ± 0.16 mm, *p* < 0.001). Angular deviation and the apico-coronal apex component did not differ significantly between BES and DE (*p* = 0.307 and *p* = 0.055).

### Effect of individual tooth position

**Fig. 6:**
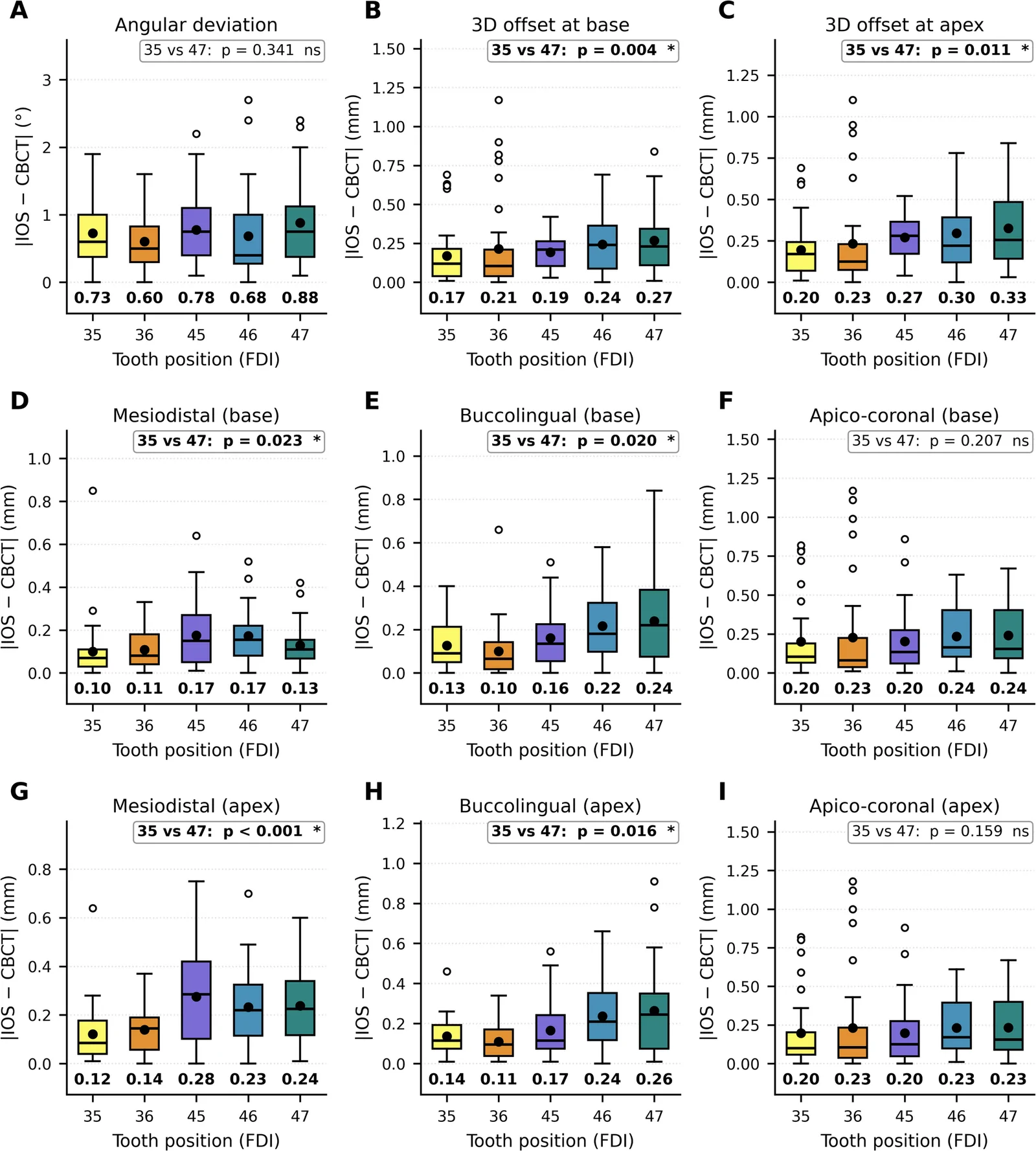
Absolute IOS–CBCT differences by tooth position according to the Fédération Dentaire Internationale (FDI) notation (35 and 36, BES; 45–47, DE; *n* = 36 per position) for all nine parameters (**A**–**I**). Values below the boxes are means. Insets give the pairwise comparison of positions 35 and 47 (Mann-Whitney U test; *, *p* < 0.05; ns, not significant). Box plot elements as in Fig. 3. BES, bounded edentulous space; DE, distal extension; CBCT, cone-beam computed tomography; IOS, intraoral scan; 3D, three-dimensional

When deviations were analyzed by FDI position (Fig. 6), a gradient from anterior to distal posterior sites was apparent for the 3D offsets, which were smallest at position 35 and largest at position 47: in the pairwise comparison of these positions, the 3D base offset increased from 0.17 mm to 0.27 mm (*p* = 0.004) and the 3D apical offset from 0.20 mm to 0.33 mm (*p* = 0.011). A direct comparison of the homologous positions 35 (BES) and 45 (DE) showed significantly larger differences at position 45 for the 3D offsets (base *p* = 0.033; apex *p* = 0.005) and the mesiodistal components (base *p* = 0.005; apex *p* < 0.001), consistent with the overall BES-vs-DE contrast. Within the matched-position comparison of 36 (BES) and 46 (DE), five of nine parameters differed significantly in the same direction as the overall BES-vs-DE contrast, including the buccolingual components at the implant base (*p* < 0.001) and apex (*p* < 0.001), suggesting clinical indication rather than side or absolute position as the dominant driver. The position-dependent increase therefore largely paralleled the BES-to-DE transition along the dental arch.

### Synthesis

Taken together, IOS- and CBCT-based three-dimensional evaluation of implant positioning yielded small differences with only minor systematic bias across the full sample, with limits of agreement within the order of one millimeter and approximately two degrees. Within-method reproducibility was superior for IOS in eight of nine parameters, and IOS variability was predominantly driven by scan-to-scan rather than within-scan variation. Discrepancies between modalities were not uniformly distributed but increased with progression from bounded to free-end situations and with more distal positions, suggesting clinical configuration rather than modality choice per se as a principal determinant of paired-measurement disagreement.

## Discussion

### Principal findings

This paired in vitro method-comparison study quantified agreement and within-method reproducibility of IOS- and CBCT-based three-dimensional implant position assessment in the posterior mandible. Three principal observations emerged. First, paired IOS–CBCT differences were small, with only minor systematic bias, across all nine geometric parameters, with 95% limits of agreement on the order of two degrees angular and one millimeter linear deviation (Figs. 2 and 3). Second, within-method reproducibility, expressed as the within-implant standard deviation Sw, was superior for IOS in eight of nine parameters, and the dominant source of IOS variability was the scan acquisition step rather than repeated digital re-evaluation of the same scan (Fig. 4). Third, between-modality disagreement was not uniformly distributed: it increased systematically from bounded edentulous to distal extension sites (Fig. 5) and from anterior to more distal posterior positions (Fig. 6), suggesting clinical configuration as the principal determinant of paired-measurement disagreement.

### Comparison with the current evidence base

The most recent systematic review and meta-analysis on this question, including 14 studies, reported pooled IOS–CBCT mean differences of 0.27 mm (95% confidence interval [CI] 0.13–0.42) at the implant platform, 0.22 mm (95% CI 0.14–0.30) at the apex and 1.24° (95% CI 0.48–1.99) for angular deviation, with no significant difference between methods in studies that measured implant deviation by both modalities [19]. The mean absolute differences observed in the present sample (0.22 ± 0.20 mm at the base, 0.26 ± 0.22 mm at the apex, 0.73° ± 0.55° for the angle) lie within or below those pooled estimates, situating the present data well within the contemporary range. However, the GRADE *(Grading of Recommendations Assessment*,* Development*,* and Evaluation)* certainty for the underlying clinical evidence was rated as “very low” in that meta-analysis, reflecting both the limited number of in vivo studies and the heterogeneity of measurement frameworks — a gap that the present standardized paired design with formal Bland-Altman and Sw analyses is intended to help address.

Within the directly comparable primary literature, the overall magnitude of paired differences is consistent across study types. Mean angular deviations in the present sample fall in the same range as values reported by in vitro work using comparable digital workflows (0.62°–0.74°) [8] and 0.72° ± 0.52° [20]. Three-dimensional offsets at the implant base (0.22 ± 0.20 mm) and apex (0.26 ± 0.22 mm) overlap closely with the corresponding values reported by Zhou and colleagues (0.23 ± 0.13 mm and 0.27 ± 0.14 mm) [20], with prior in vitro estimates of 0.12–0.14 mm [8], and with 0.03 ± 0.17 mm and 0.04 ± 0.22 mm reported using an alternative scanner–software combination [21]. Apico-coronal deviations of 0.22 ± 0.23 mm are in line with values of 0.19–0.21 mm in comparable in vitro work [8], while directional analyses in the mesiodistal and buccolingual planes parallel those from in vivo investigations [12].

Recent in vivo evidence reinforces this picture. A prospective clinical cohort using digital registration of IOS data against postoperative CBCT reported overall root-mean-square deviations of 0.29 ± 0.05 mm across single and dual implants, with no significant difference between configurations [13]. A cross-sectional clinical study on robotic-assisted implant surgery in posterior partial edentulism reported essentially identical IOS-based and CBCT-based deviation estimates (global coronal 0.59 ± 0.23 vs. 0.59 ± 0.24 mm; global apical 0.66 ± 0.24 vs. 0.63 ± 0.25 mm; angular 1.28° ± 0.69° vs. 1.19° ± 0.70°), with intra-class correlation coefficients (ICCs) of 0.91 (coronal), 0.89 (apical) and 0.86 (angular) and Bland-Altman analyses showing excellent agreement [22]. An earlier in vitro paired analysis using the same superimposition framework reported ICCs of 0.99 across all three parameters and Bland-Altman limits of agreement comparable to those reported here [23]. Larger laboratory-scanner setups have produced systematically smaller mean differences, in the order of hundredths of a millimeter [23, 24], reflecting differences in setup standardization rather than a fundamentally different underlying agreement structure. Adjacent meta-analytic work on the wider digital implant workflow [25, 26] confirms the same overall direction: clinically small and unbiased IOS–CBCT differences in single and short-span partial edentulism, with rising heterogeneity as edentulous extent or anatomical complexity increases.

The present analysis adds two elements that the current evidence base has not addressed. First, none of the cited primary studies or meta-analyses, to our knowledge, has decomposed within-method variability into within-scan and between-scan components, suggesting that the present work may be among the first to identify scan acquisition rather than re-evaluation as the predominant source of IOS dispersion. Second, the systematic stratification by indication (BES vs. DE) and by individual FDI position within a single, internally consistent posterior-mandibular dataset goes beyond the aggregate “single-tooth” or “short-span” comparisons that dominate the current literature [19, 27].

### Mechanistic interpretation

The position- and indication-dependent gradient observed in the present data has a plausible geometric rationale. Both modalities ultimately register the implant against the surrounding anatomy: IOS via optical referencing to neighboring tooth surfaces, CBCT via landmark-based registration of the volumetric datasets. In bounded edentulous spaces, scan bodies are flanked by intact occlusal, vestibular and oral tooth surfaces on both sides, providing dense and unambiguous geometric anchors for both the optical scan stitching and the subsequent superimposition with the preoperative CBCT. As edentulous distance increases towards distal extension sites and the most posterior FDI positions, fewer adjacent reference surfaces remain available distally; optical scan stitching has to bridge longer surface-free segments, and the matching algorithm has fewer common features to align CBCT and STL datasets. In addition, the three registration landmarks (cusp tips of 37, 34 and 44) enclose the bounded edentulous space, whereas the distal extension sites lie distal to the landmark-defined reference area; small rotational registration errors may therefore translate into larger linear discrepancies with increasing distance from the landmarks, a plausible but not directly tested lever-arm effect. The greater paired-measurement disagreement at positions 36 versus 46 and the overall increase from position 35 to 47 are consistent with this geometric explanation and reproduce previously reported scanner-distance and scan-span effects in fully digital workflows [1, 27, 28]. In contrast, angular deviation, which is less sensitive to the absolute scan extent than to local scan body orientation, did not differ significantly between gap types, in agreement with prior reports on angulation-related effects [29].

The pronounced reduction in IOS within-method dispersion compared with CBCT, and particularly the small within-scan but larger between-scan Sw, suggests that for a given acquired scan the digital evaluation step is highly stable, whereas successive intraoral acquisitions remain the principal source of variability. This pattern is compatible with reports that scanner positioning, ambient lighting and scan trajectory shape the precision of intraoral scans more than the subsequent measurement step [11, 30]. From a measurement-theory perspective, the present Sw values for IOS may approach the spatial resolution of the underlying scan-body geometry, while CBCT Sw values likely reflect both the voxel resolution of the imaging device and the manual marker placement in apico-coronal direction, which has previously been identified as a substantial source of CBCT measurement uncertainty [3].

### Methodological considerations for the two modalities

Both modalities have intrinsic resolution and reproducibility limits that are relevant for the interpretation of the present results. For CBCT, spatial resolution is co-determined by voxel size, field of view, exposure parameters and reconstruction, and metallic implants generate artifacts that impair the delineation of implant contours [9]. The voxel size of 0.3 mm used here lies within the range considered adequate for linear measurements in implant planning [31], but it defines a resolution limit that is considerably coarser than that of optical surface acquisition. Together with the manual, operator-dependent definition of the implant axis in the presence of metal artifacts [3, 9], this offers a plausible explanation for the higher within-method variability of the CBCT-based evaluation observed here.

For IOS, the present results are specific to the scanner used. In comparative benchmark studies, TRIOS 4 has shown trueness comparable to that of other current devices over short distances, but not the highest precision, and the precision of intraoral scanners generally decreases with increasing inter-implant distance [32]. The IOS reproducibility values reported here should therefore be regarded as characteristic of this scanner and scan-body combination rather than of IOS as a category; devices with higher precision may yield lower between-scan variability [32, 33].

Finally, the lower agreement in distal extension sites is consistent with the known dependence of intraoral scanning accuracy on adjacent tooth-supported reference structures and on scan span [27, 28]. A recent clinical study of 278 implants likewise reported closer agreement between IOS- and CBCT-based postoperative assessment in tooth-bounded than in free-end situations, with apical and vertical components contributing most to the differences [34]. The convergence of these in vivo findings with the present in vitro data supports the interpretation that the edentulous configuration is a principal determinant of paired-measurement disagreement.

### Clinical implications

Within the limits of an in vitro design, the present results support a differentiated use of IOS-based superimposition as a radiation-free complement, rather than a general replacement, for postoperative three-dimensional implant position assessment in the posterior mandible. For single implants placed within bounded edentulous posterior sites, paired-measurement differences and IOS within-method reproducibility were both within ranges generally considered clinically acceptable, and the limits of agreement remained substantially below the safety margins recommended for adjacent anatomical structures in implant planning [2, 35]. This is consistent with the favorable agreement reported for digital registration approaches in single- and dual-implant clinical cohorts [13, 22]. In this configuration, and pending confirmation in vivo, IOS-based assessment may be sufficient for routine follow-up and may help to operationalize the ALARA principle by avoiding additional radiation exposure when there is no specific clinical indication for CBCT control imaging [6, 36]. The present data do not, however, support a routine replacement of CBCT in all clinical situations.

In distal extension situations and at the most posterior positions, by contrast, paired-measurement disagreement increases in seven of nine parameters, with individual absolute apical differences approaching the millimeter range. In these configurations, IOS-based assessment should not be interpreted as if its precision were spatially uniform across the dental arch. Whenever the postoperative measurement is intended to drive a clinical decision that depends on sub-millimeter or sub-degree precision — for example, proximity assessment to the inferior alveolar canal, prosthetic axis verification or surgical re-intervention planning — a complementary CBCT-based control remains advisable. The same applies when the clinical question is framed against safety margins that approach the order of magnitude of the observed limits of agreement.

Beyond the immediate workflow choice, the variance decomposition has a direct practical implication: efforts to further improve IOS-based assessment should preferentially target the scan acquisition step (operator technique, scan strategy, scan body design, lighting) rather than the digital re-evaluation [27, 30, 33].

### Strengths and limitations

The principal strength of the present analysis is the paired design with repeated IOS- and CBCT-based assessments of every implant, enabling formal quantification of both between-modality agreement and within-method reproducibility, including the decomposition of IOS variability into within-scan and between-scan components. The clinically simulated setup, with a patient-simulation unit and a facial mask, represents an intermediate position between idealized phantom studies and uncontrolled in vivo conditions and has been described previously for the same sample [18].

Several limitations should be acknowledged. First, the analysis is based on a previously reported in vitro platform [18]; intraoral confounders such as saliva, bleeding, patient movement and variable muscular soft-tissue support are not represented [28]; in addition, no gingival mask was used. Second, only a single mandibular model, a single intraoral scanner system (TRIOS 4, 3Shape) and a single scan-body geometry (Straumann CARES RC) were investigated; other devices, scan-body designs and post-sterilization conditions may yield different results [30, 32, 33, 37]. Third, the examiners performing the evaluations were not blinded to the imaging modality, and no formal washout period separated the repeated evaluations, leaving residual potential for recall bias; this caveat is shared with most comparable workflow studies [3]. Fourth, no true reference standard for in-situ implant position was available; consequently, the analysis is framed in terms of paired agreement and within-method reproducibility rather than as a determination of which modality more closely reflects the true implant position — a framing that converges with the methodological caution recently emphasized in the field [19, 26]. Fifth, implant angulations were not systematically varied, although tilted distal implants — for example in all-on-four configurations — may behave differently with respect to paired-measurement agreement [29]. Sixth, the present sample is restricted to the posterior mandible with one defined dental configuration; extrapolation to fully edentulous arches, anterior regions, the maxilla and to multi-implant restorations requires dedicated investigation [8, 27, 38]. Seventh, only one postoperative CBCT was acquired per model, so that CBCT Sw reflects evaluation variability only, whereas IOS Sw additionally includes re-acquisition; moreover, the Treatment Evaluation Tool reports unsigned deviation magnitudes relative to the plan rather than implant coordinates, and landmark-based registration errors cannot be entirely excluded despite visual verification. Finally, repeated assessments of the same implants and models were analyzed as individual observations; the p values of the subgroup comparisons should therefore be regarded as exploratory.

Future work should therefore extend the present approach to in vivo cohorts, additional anatomical regions, multiple scanner systems and varying implant angulations, ideally combined with blinded evaluation and explicit reference-free reproducibility analyses, so that the indication- and position-specific behavior of IOS-based postoperative assessment can be characterized across the relevant range of clinical situations.

## Conclusion

In this paired in vitro method-comparison study of posterior mandibular implant sites, IOS- and CBCT-based three-dimensional assessment showed small differences with only minor systematic bias, with limits of agreement on the order of two degrees angular and one millimeter linear deviation. Within-method reproducibility was superior for IOS in eight of nine parameters, with scan acquisition rather than digital re-evaluation identified as the predominant source of variability. Both indication and FDI tooth position modulated paired-measurement disagreement, which increased from bounded edentulous to distal extension sites and from anterior to more distal posterior positions.

Within the limits of this in vitro design and pending confirmation in vivo, IOS-based superimposition may be considered a radiation-free complement to CBCT for the postoperative monitoring of single implants in bounded edentulous posterior sites, rather than a general replacement. In distal extension situations and at the most posterior positions, paired-measurement disagreement increases; in such configurations IOS-based assessment should be interpreted with greater caution, and a complementary CBCT-based control remains advisable whenever clinical decision-making depends on sub-millimeter or sub-degree precision. Within this differentiated framework, further methodological gains are most likely to be achieved by optimizing the scan acquisition step rather than the subsequent digital evaluation.

## Data Availability

The datasets generated and/or analyzed during the current study are available from the corresponding author on reasonable request.
